# Epithelial and Stromal Cell Urokinase Plasminogen Activator Receptor Expression Differentially Correlates with Survival in Rectal Cancer Stages B and C Patients

**DOI:** 10.1371/journal.pone.0117786

**Published:** 2015-02-18

**Authors:** Seong Beom Ahn, Charles Chan, Owen F. Dent, Abidali Mohamedali, Sun Young Kwun, Candice Clarke, Julie Fletcher, Pierre H. Chapuis, Edouard C. Nice, Mark S. Baker

**Affiliations:** 1 Australian School of Advanced Medicine, Faculty of Human Science, Macquarie University, North Ryde, NSW 2109, Australia; 2 Anatomical Pathology Department, Concord Hospital and Discipline of Pathology, University of Sydney, Concord, NSW 2139, Australia; 3 Department of Colorectal Surgery, Concord Hospital and Discipline of Surgery, University of Sydney, Concord, NSW 2139, Australia; 4 Department of Biochemistry and Molecular Biology, Monash University, Clayton, VIC 3800, Australia; Queen Mary Hospital, HONG KONG

## Abstract

Urokinase plasminogen activator receptor (uPAR) has been proposed as a potential prognostic factor for colorectal cancer (CRC) patient survival. However, CRC uPAR expression remains controversial, especially regarding cell types where uPAR is overexpressed (e.g., epithelium (uPAR^E^) or stroma-associated cells (uPAR^S^)) and associated prognostic relevance. In this study, two epitope-specific anti-uPAR monoclonal antibodies (MAbs) could discriminate expression of uPAR^E^ from uPAR^S^ and were used to examine this association with survival of stages B and C rectal cancer (RC) patients. Using immunohistochemistry, MAbs #3937 and R4 were used to discriminate uPAR^E^ from uPAR^S^ respectively in the central and invasive frontal regions of 170 stage B and 179 stage C RC specimens. Kaplan-Meier and Cox regression analyses were used to determine association with survival. uPAR expression occurred in both epithelial and stromal compartments with differential expression observed in many cases, indicating uPAR^E^ and uPAR^S^ have different cellular roles. In the central and invasive frontal regions, uPAR^E^ was adversely associated with overall stage B survival (HR = 1.9; p = 0.014 and HR = 1.5; p = 0.031, respectively) reproducing results from previous studies. uPAR^S^ at the invasive front was associated with longer stage C survival (HR = 0.6; p = 0.007), reflecting studies demonstrating that macrophage peritumoural accumulation is associated with longer survival. This study demonstrates that different uPAR epitopes should be considered as being expressed on different cell types during tumour progression and at different stages in RC. Understanding how uPAR^E^ and uPAR^S^ expression affects survival is anticipated to be a useful clinical prognostic marker of stages B and C RC.

## Introduction

Recent data from the World Health Organisation indicates colorectal cancer (CRC) is the third most common malignancy (~1.36 million cases worldwide in 2012) with a mortality of over 50% [1]. The major cause of cancer related death is metastasis. Clinico-pathological staging of CRC demonstrates a dramatic fall in survival between stages B and C, corresponding to absence versus presence of lymph node metastasis [2]. Despite its clinical relevance, the molecular mechanisms underpinning metastasis are still not fully characterised and development of new targeted strategies to counter metastasis remain elusive.

The plasminogen activation proteolytic cascade is one of a number of pivotal biological processes implicated in cancer cell invasion and metastasis. These include extracellular matrix (ECM) degradation allowing detachment of tumour cells from the original site and penetration of basement membrane, growth factor activation and intracellular signalling [3]. A glycosylphosphatidylinositol-anchored membrane protein called urokinase plasminogen activator receptor (uPAR) is central to this cascade. uPAR is a tri-domain protein (i.e., D1, 2 and 3) which forms a thick-fingered glove-like receptor providing a central pocket for the binding of its cognate protease ligand, urokinase plasminogen activator (uPA) [4]. Initial studies focused on the regulation of proteolysis (i.e., plasminogen and MMP activation) though uPAR. More recently, it has been shown that up to 42 proteins (9 extracellular and 33 lateral interacting partners) purportedly interact with uPAR [5]. The shape of uPAR involves a large contralateral external surface which is suggested to facilitate interaction/s with many of these ancillary proteins [4]. This large repertoire of interactions suggests that uPAR has evolved a complex regulatory mechanism to control proteolysis, cell migration, proliferation, cell signalling and other aspects of cell behaviour. In fact, in the last decade, extensive evidence has shown uPAR is implicated in cell adhesion, proliferation, migration, tissue remodelling and in the regulation of signalling pathways (e.g., MAP kinase, Ras pathways) [3]. These are important features not only of ubiquitous developmental pathways, but also cancer metastasis.

uPAR expression in various cancers has been extensively studied over the past two decades, as reflected by >800 uPAR oncology-related publications [6]. However, uPAR expression in the cancer microenvironment remains controversial, in particular with regard to the cell type/s on which uPAR is overexpressed (e.g., uPAR expression in epithelia (uPAR^E^) or stroma-associated cells (uPAR^S^)) [6,7]. Association between uPAR and cancer was first recognised in 1991 [8]. Since then, numerous studies have evaluated the levels of uPAR^E^ and uPAR^S^ in various cancers using an extensive range of antibodies [6,7]. However, there have been conflicting results. Specifically in CRC, Pyke *et al*., found that uPAR was strongly expressed in tumour-infiltrating macrophages, neutrophils and eosinophils (using immunohistochemistry (IHC)) but only weakly to moderately expressed in neoplastic tumour cells (using monoclonal antibodies (MAbs) against human uPAR clones R2 and R4) [9]. Later, another study reported that uPAR expression occurred mainly in tumour epithelia rather than stroma (using the anti-uPAR MAb #3937) [10]. Despite this apparent contradiction, both studies agreed uPAR was highly expressed in the tumour microenvironment and was concentrated at the tumour invasive front. Further studies on uPAR^E^ and uPAR^S^ in CRC [6,7,11–17] generally agreed that high uPAR^E^ is independently and adversely related to patient survival [11,12,15]. Seetoo *et al*. [12] suggested that uPAR (expressed mainly in epithelia) is an independent predictor of liver metastasis and overall patient survival post CRC resection. In agreement, a more recent study showed significantly elevated uPAR in CRC tumours at infiltrating tumour margins which was associated with poorer survival [15]. However, data on uPAR^S^ in CRC are contradictory in terms of survival association. It has been suggested that macrophages, which are a major source of uPAR in stroma, play a role in preventing haematogenous metastasis [16]. Additionally, an inverse association between CRC liver metastasis and uPAR primary tumour stromal expression was observed [18]. Whilst not directly correlating uPAR^S^ with patient survival, it is well-known that CRC patients with liver metastasis have significantly shorter survival than those without. In contrast, a recent report suggested that both uPAR^E^ and uPAR^S^ were negatively associated with overall CRC patient survival, as well as with disease free survival (DFS) [17]. However, only uPAR^S^ was independently associated with DFS in multivariable analysis. Collectively, we propose that these conflicting observations are due to the use of particular MAbs with different uPAR epitope-specificity when uPAR is involved in cell-specific protein interactions. In fact, most uPAR^E^ or uPAR^S^ studies were performed with a single MAb and hence will only detect specific uPAR domain/s. Previous studies have demonstrated that uPAR can be present in either full length, soluble and/or cleaved domain forms and these may be differentially expressing epitopes identified by specific MAbs [19]. Additionally, some epitopes may be masked when uPAR interacts with ancillary proteins. Therefore, to precisely examine the prognostic relevance of uPAR in cancer, MAbs detecting different key epitopes expressed on particular cell types should be applied to identical pathological tissue samples.

In this study, taking account of questions regarding uPAR expression on different cell types, we specifically measured uPAR^E^ and uPAR^S^ across a cohort (n = 349) of non-metastatic (stage B) and nodal-metastatic (stage C) rectal cancer (RC) specimens. Two commercially available epitope-specific anti-uPAR MAbs #3937 (for uPAR^E^) and R4 (for uPAR^S^) were used to probe serial sections of RC tissue microarrays (TMA). Within each sample, uPAR^E^ and uPAR^S^ were measured in the central region, the invasive tumour front and adjacent non-neoplastic mucosa. The aim was to assess associations between uPAR measurements and pathological characteristics of the tumour and patients’ overall survival.

## Materials and Methods

### Patient cohort and tumour characterisation

Data were drawn from a prospective registry of consecutive colorectal cancer resections which was initiated in 1971 at Concord Hospital, a public tertiary referral hospital in Sydney, Australia and contains detailed clinical, operative, pathology, adjuvant therapy and follow-up information [20,21]. All resections were performed by specialist colorectal surgeons using a standardized technique [22]. Resections for rectal cancer between 1988 and 2001 inclusive were selected for analysis and all non-deceased patients were followed for a minimum of five years. Only adenocarcinomas were included in the registry. Where multiple tumours were present, only the most advanced-stage lesion was included. Patients were excluded if they had previous colorectal cancer, inflammatory bowel disease or familial adenomatous polyposis coli. The rectum was defined as including the rectosigmoid junction but excluding the anal canal. Over 90% of specimens were examined according to a standard protocol [2] by a single pathologist (R.C. Newland) who also reviewed the remainder. Tumour size was measured as the greatest surface dimension and blocks were taken to demonstrate maximum direct tumour penetration of the bowel wall. Additional blocks were taken to demonstrate the relationship between tumour and any adherent structure or tissue as well as lines of resection and the free serosal surface. Venous invasion, assessed by hematoxylin and eosin staining, was recorded as involvement of thick or thin-walled veins, either within or beyond the bowel wall. When doubt existed as to whether a structure involved was a vein, a negative finding was recorded. An apical lymph node was defined as the most proximal node found within 1 cm of the vessel ligation at the apex of a vascular pedicle. Tumours were histologically classified as low-, average-, or high grade malignancy. Grade was assessed taking into account the degree of differentiation and anaplasia, the nature of the tumour margin (pushing or infiltrating) and the presence and prominence of vascular invasion. In advanced stage tumours the proportion of involved lymph nodes was calculated as a percentage of the total number of nodes harvested. Before 2002 over 90% of specimens were reported on or reviewed by a single pathologist (R.C. Newland). All pathology features analysed were looked for in every specimen and their presence or absence recorded explicitly. There were no missing data on any variable. Tumours were staged according to the Australian Clinico-Pathological Staging (ACPS) system for CRC [2]. The four main stages of this system (A, B, C, D) are directly equivalent to the main stages (I, II, III, IV) of the pTNM system [23] but, importantly, ACPS differs in that all lesions with macro- or microscopic tumour in any resection margin are coded as stage D. The CRC registry at Concord Hospital is conducted with the approval of the South Western Sydney Health Area Ethics Committee (CH62/6/2011–136) with written consent in accordance with the requirements of the NSW Human Tissue Act 1983 and the NHMRC National Statement on Ethical Conduct in Human Research 2007. The study was also approved by the Macquarie University Human Ethics Committee (#5201100858).

### TMA construction

Formalin-fixed paraffin-embedded TMA were constructed using an Advanced Tissue Arrayer ATA-100 (Chemicon, Temecula, CA, USA). From original rectal cancer tissue paraffin blocks, cores (1.5mm) were taken from selected morphologically representative areas of central region of the tumour (avoiding luminal surfaces), the invasive front of the tumour and histologically normal mucosa (1–2cm from the tumour margin) and arrayed into freshly made recipient paraffin blocks.

### IHC

TMA sections were prepared and processed simultaneously with the same batches of antibodies and reagents, and staining was performed in a single clinical pathology laboratory on a single run of all samples. Two epitope-specific murine anti-human uPAR MAbs, #3937 (American Diagnostica Inc (ADI), Greenwich, CT, USA) and R4 (Dako, Glostrup, Denmark), were used for IHC. Staining was performed with a polymer-based IHC detection system on a Bond-Max Autostainer (Leica Biosystems, Melbourne, Australia) as described [24] with some modifications. For #3937 MAb staining, no antigen retrieval was performed and 3.33µg/ml of #3937 was applied to TMA sections. For R4 MAb staining, proteinase K (Leica Microsystems) was used for antigen retrieval, the concentration of R4 being 10µg/ml. Negative control slides were incubated with isotype IgG1 (R&D Systems, Minneapolis, USA) using the same antigen retrieval methods and concentrations as used for both primary antibodies.

### IHC evaluation

Staining intensities for both MAbs were evaluated independently by two assessors (SBA, CC), who were blinded to patients’ clinico-pathological status. Scoring was performed separately for the central region, invasive tumour front and adjacent non-neoplastic mucosa: 0 = none, 1 = weak, 2 = moderate and 3 = strong staining. The concordance rate between both assessors across the whole group of samples was over 95% and any disagreement/s were resolved by joint re-examination of the data, discussion and re-review of scores. If staining intensity was heterogeneous in any single tissue core, the predominant staining intensity was recorded.

### Outcome variable and patient follow-up

Overall survival time was measured from date of surgical resection to date of death, with times censored for patients lost to follow-up or who remained alive at study close. Patients were followed annually until death or up to December 31, 2011. The follow-up protocol has been described elsewhere [22].

### Statistical Analysis

Chi-squared χ² test or Fisher’s exact test were used to examine statistical significance of differences in proportions. The Wilcoxon matched pairs signed ranks test was used to compare the frequency distributions of uPAR^E^ and uPAR^S^ between the central region and invasive tumour front. Comparisons of overall survival time between strata of uPAR expression and covariates were made with the Kaplan-Meier method and log-rank test and also Cox regression and Wald test. Continuous and multi-category covariates were dichotomised at conventional or otherwise appropriate cutting points. As the clinico-pathological stage is the strongest known predictor of prognosis, associations with overall survival were examined for stages B and C separately as well as for combined stages, in order to identify any differences in effects of uPAR^E^ and uPAR^S^ between stages. The level for two-tailed statistical significance was p≤0.05 with confidence intervals at the 95% level. Analyses were performed with SPSS version 20 (IBM Australia Limited).

## Results

Between January 1988 and December 2001 there were 782 rectal cancer resections; 206 for stage B and 251 for stage C tumour. Resection specimens for 77 (31 from stage B and 46 from stage C) did not yield sufficient or appropriate archival material for TMA construction. The remaining 175 stage B and 205 stage C specimens yielded informative IHC results that varied by stage, site and for uPAR^E^, uPAR^S^ and uPAR^PT^ (peritumoural uPAR) expression. After excluding those with no informative IHC, there remained 170 patients with stage B and 179 with stage C tumour for whom data were available on at least one of central or frontal uPAR^E^, uPAR^S^ or uPAR^PT^. Clinical and pathology features of these patients are shown in Table 1. For stages B and C separately and for the uPAR assessments separately we compared patients who had a TMA/IHC result with those lacking a result in regard to the variables in Table 1 and found only three statistically significant differences (data not shown), from which we concluded that the patients with a TMA/IHC result whom we analysed for this study did not differ materially from the excluded patients who had no TMA/IHC result. In subsequent tables the numbers of patients analysed vary according to the number who had to be excluded because of missing IHC results.

**Table 1:** Clinical and pathology features of 170 patients with stage B tumour and 179 with stage C tumour for whom data were available for either central or frontal uPAR^E^, uPAR^S^ or uPAR^PT^. Number (percent).

Variable	Category	Stage B n = 170	Stage C n = 179
Sex	Male	113 (67)	113 (63)
	Female	57 (33)	66 (37)
Age	20–74 years	122 (72)	132 (74)
	≥ 75 years	48 (28)	47 (26)
Urgent resection	Yes	1 (1)	3 (2)
	No	169 (99)	176 (98)
Type of surgery	Anterior resection	124 (73)	145 (81)
	Abdominoperineal excision	33 (19)	25 (14)
	Hartmann’s operation	13 (8)	9 (5)
Tumour distance from anal verge	< 7 cm	61 (36)	50 (28)
	8–12 cm	60 (35)	74 (41)
	> 12 cm	49 (29)	55 (31)
Tumour maximum surface dimension	< 5 cm	72 (42)	102 (57)
	≥ 5 cm	98 (58)	77 (43)
Histological type of tumour	Adenocarcinoma	164 (97)	164 (92)
	Mucinous or signet ring adenocarcinoma	6 (3)	15 (8)
Direct tumour spread	Confined to submucosa	–	5 (3)
	Not beyond muscularis propria	–	24 (13)
	Beyond muscularis propria	170 (100)	150 (84)
Number of lymph nodes involved	None (N0)	–	–
	1–3 (N1)		116 (65)
	>3 (N2)		63 (35)
Percent of involved nodes	< 40%	–	141 (79)
	≥ 40%		38 (21)
Apical node involved	Yes	–	9 (5)
	No		170 (95)
Tumour grade	Low	19 (11)	1 (1)
	Average	127 (75)	111 (62)
	High	24 (14)	67 (37)
Free serosal surface involved	Yes	5 (3)	15 (8)
	No	165 (97)	164 (92)
Venous invasion	Yes	33 (19)	63 (35)
	No	137 (81)	116 (65)
Adjacent organ infiltrated	Yes	2 (1)	6 (3)
	No	168 (99)	173 (97)
Preoperative radiotherapy with or without chemotherapy	Yes	7 (4)	8 (5)
	No	163 (96)	171 (95)
Postoperative radiotherapy	Yes	6 (3)	3 (2)
	No	164 (97)	176 (98)
Postoperative chemotherapy	Yes	5 (3)	45 (25)
	No	165 (97)	134 (75)

### Detection and comparison of uPAR^E^ and uPAR^S^ in RC

The main aim of the present study was to correlate uPAR^E^ and uPAR^S^ with patient survival in stages B and C RC. To achieve this, we initially determined the location and expression levels of uPAR^E^ and uPAR^S^ in RC tissues using two epitope-specific anti-uPAR MAbs #3937 and R4. These MAbs were chosen since #3937 and #3936 (both from ADI) were the most common commercially available MAbs used for uPAR^E^ detection, and R4 & R2 (from Dako and/or collaborators at Finsen Laboratory) were most commonly used to differentiate uPAR^S^ [6,7,9,10]. MAb #3936 was also tested to detect uPAR^E^ in our study: however we were not able to optimise a reliable antigen retrieval method for consistent detection of uPAR^E^ using this MAb. Specifically MAb #3936 gave high background staining or no staining depending upon the antigen retrieval methods used and, more importantly, an isotype control (IgG2a) also weakly stained the RC TMA (data not shown). Isotype control MAbs for #3937 and R4 (i.e., IgG1) were also tested with relevant antigen retrieval methods and no non-specific binding was observed (data not shown). R2 was not used in this study.

**Fig 1:**
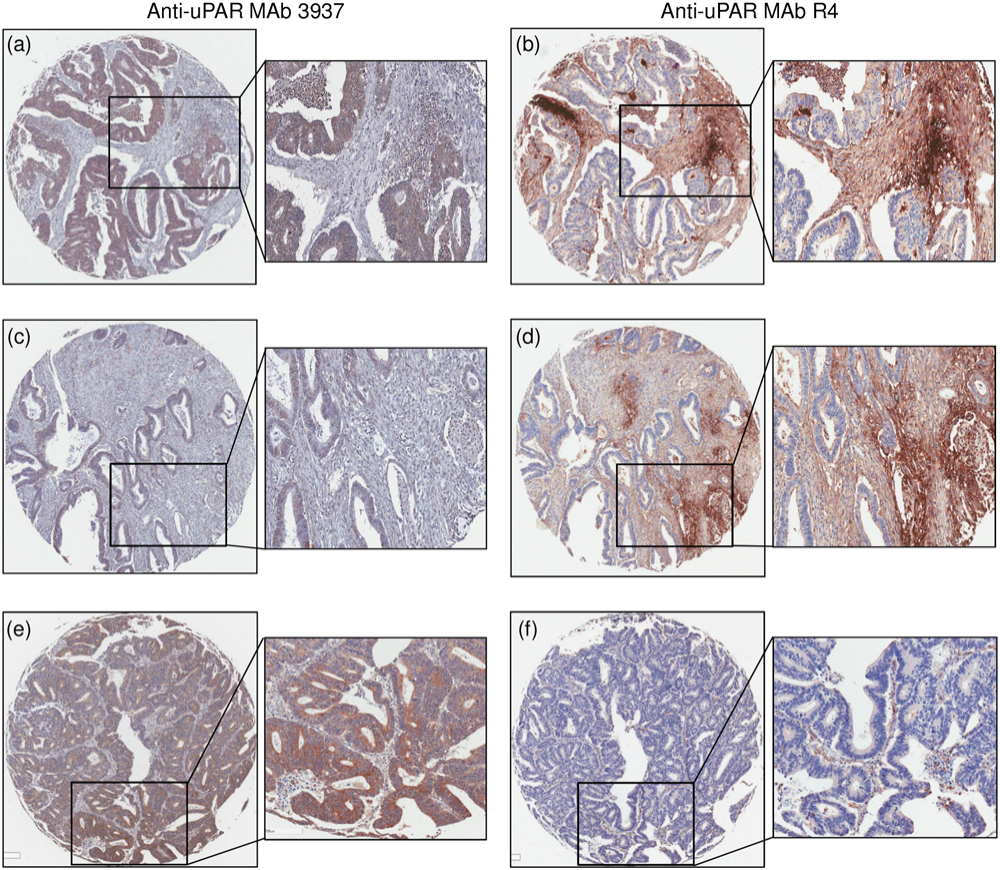
Expression of uPAR^E^ and uPAR^S^ in rectal cancer tissue microarray, detected by different epitope-specific MAbs: 3937 (left column) and R4 (right column). Images in rows (i.e., (a) & (b) or (c) & (d) or (e) & (f)) are the same tissue cores from serial sections of the tissue microarray. (a) & (b) represent strong expression of both uPAR^E^ and uPAR^S^. (c) & (d) exemplify partial expression of uPAR^E^ and strong uPAR^S^, respectively. Conversely, (e) & (f) show strong uPAR^E^ and partial uPAR^S^, respectively.

**Fig 2:**
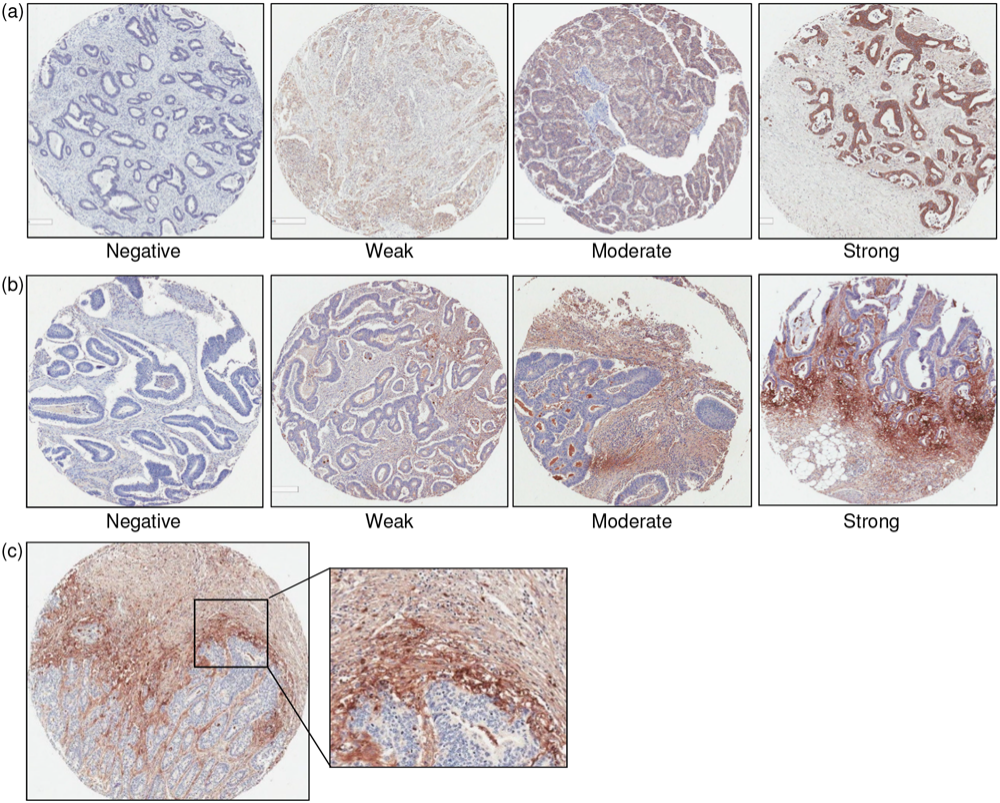
Staining intensities of uPAR^E^ (a; stained with anti-uPAR #3937 MAb) and uPAR^S^ (b; stained with anti-uPAR R4 MAb) were evaluated as negative, weak, moderate or strong. Peritumoral accentuation of uPAR (i.e., uPAR^PT^; uPAR expression in stroma-associated cells and concentrated around the tumour epithelium) represented in (c), stained with R4.

IHC demonstrated that MAb #3937 primarily stained the RC epithelia and stained some stroma-associated cells weakly. Conversely, R4 was mainly confined to RC stroma-associated cells with very weak epithelial staining (Fig. 1). Importantly, in many cases differential staining patterns of #3937 and R4 were observed using serial sections from the same TMA, indicating that uPAR^E^ and uPAR^S^ were differentially expressed. For instance, in some cases, uPAR was overexpressed in both epithelia and stroma (Fig. 1a & 1b), and in other cases uPAR was weakly expressed in epithelia but strongly expressed in stroma (Fig. 1c & 1d). The opposite was also observed (i.e., high uPAR^E^ with weak uPAR^S^; Fig. 1e & 1f). To compare the distribution between uPAR^E^ and uPAR^S^, expression levels were evaluated based on staining intensities (Fig. 2a & 2b) for both the central and invasive tumour front. In the central tumour, 33% (89/268) tissue cores had approximately equal expression of uPAR^E^ and uPAR^S^, 48% (128/268) had higher expression of uPAR^E^ and 19% (51/268) had higher expression of uPAR^S^. Comparable ratios were observed in the invasive tumour front where 31% (106/338) had similar uPAR^E^ and uPAR^S^ expression, 41% (137/338) had higher uPAR^E^ and 28% (95/338) had higher uPAR^S^.

uPAR in RC tissues was highly expressed in the invasive tumour front compared to the central region for both epithelial and stromal locations, consistent with other cancer types [6,7]. A Wilcoxon matched pairs signed ranks test showed that staining intensity of both uPAR^E^ and uPAR^S^ was significantly higher at the invasive front compared to the central tumour location (uPAR^E^, n = 255, p<0.001; uPAR^S^, n = 257, p<0.001).

### Patient follow-up

At the close of study (December 2011), of the 363 patients who had an informative TMA/IHC result on uPAR^E^ or uPAR^S^, 243 were deceased, 111 had been followed for between 103 and 246 months (median 171 months) and 9 had been lost to follow-up after a median of 56 months. Of the deceased patients, 117 died of CRC, 117 of other causes and 9 from unknown causes.

### uPAR^E^ and survival in stage B tumours

**Fig 3:**
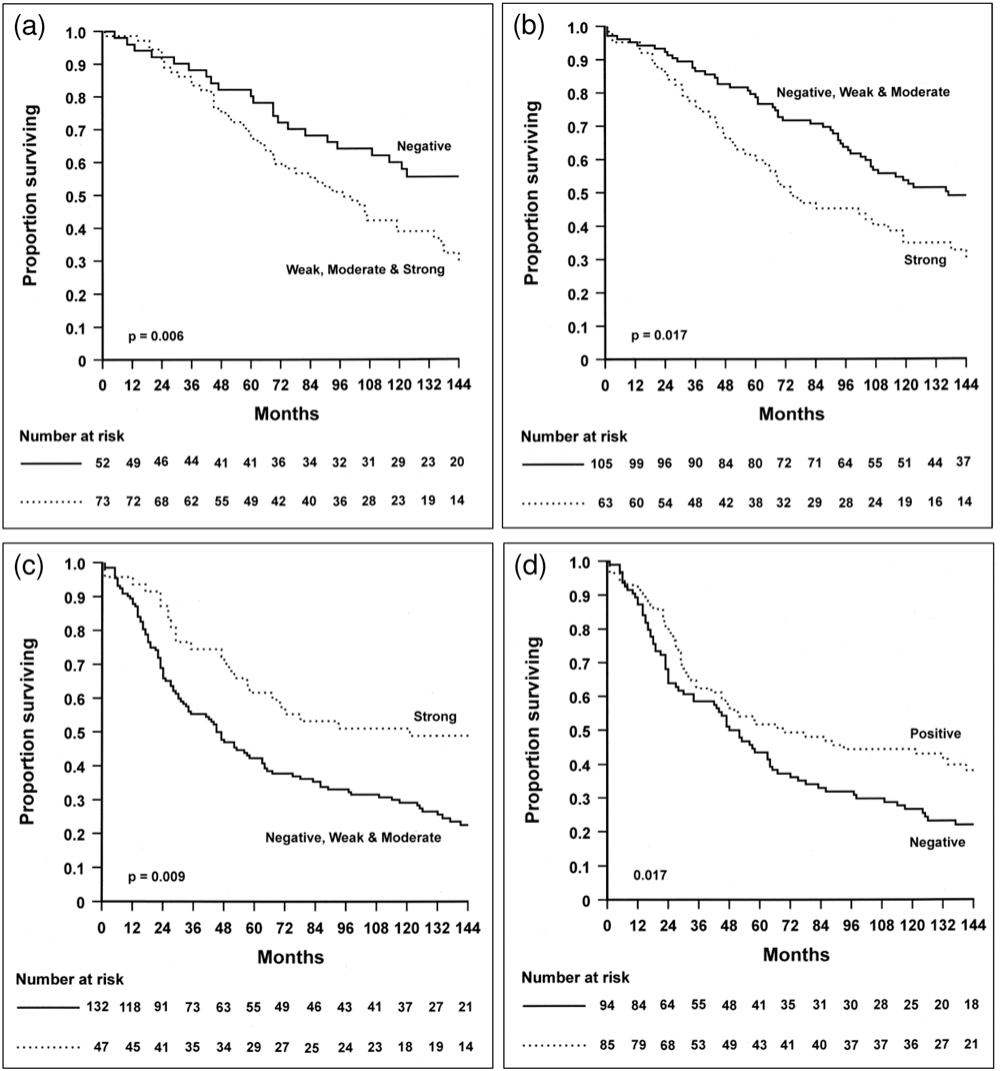
Kaplan-Meier overall survival analyses for uPAR^E^ (a and b), uPAR^S^ (c) and uPAR^PT^ (d) in rectal cancer. In stage B patients, (a): overall survival was significantly reduced with uPAR^E^ positive in central region of tumour (n = 125) and (b): with strong uPAR^E^ in invasive front of tumours (n = 168). In stage C patients, (c): strong uPAR^S^ in invasive front of tumour (n = 179) was significantly associated with longer overall survival. (d): Positive uPAR^PT^ in invasive front of tumour (n = 179) was also significantly associated with longer overall survival.

**Central region**. For stages B and C combined, stronger uPAR^E^ staining was associated with poorer survival (p = 0.004). However, when stratified by stage, this association persisted strongly for stage B (p = 0.002) but disappeared for stage C (p = 0.589). This indicates that the prognostic relevance of uPAR^E^ was confined to stage B patients. Therefore, only the association between uPAR^E^ and survival of stage B patients was analysed further. As no significant survival difference was observed between the weak and moderate staining categories, these were combined to form a single “intermediate” category. Survival was significantly poorer in the intermediate group than the negative group (p = 0.035) but not significantly different between the intermediate and strong groups (p = 0.206). Thus, the latter were combined into a single positive uPAR^E^ group. Kaplan Meier analysis showed that patients in the uPAR^E^ positive group experienced significantly poorer overall survival than those in the uPAR^E^ negative group (Fig. 3a; p = 0.006). Multivariate analysis demonstrated that uPAR^E^ in the central tumour was an independent negative prognostic indicator of overall survival in stage B RC patients after adjustment for other prognostic variables (HR 1.9 [95% CI 1.1–3.1] Wald-p 0.014) (Table 2).

**Table 2:** Association between stage B patient overall survival and expression of uPAR in tumour epithelium (detected by #3937 MAb) in both the central region and invasive front of tumour tissues with adjustment for other potentially prognostic variables.

		Stage B central region of tumour tissue (n = 125)					Stage B invasive front of tumour tissue (n = 168)				
Variable	Category	Deaths/Total	BHR (95% CI)	Wald p	MHR (95% CI)	Wald p	Deaths/Total	BAR (95% CI)	Wald p	MHR (95% CI)	Wald p
Sex	Male	44/84	0.5 (0.3–0.8)	0.006	–	–	62/112	0.7 (0.5–1.1)	0.102	–	–
	Female	31/41					39/56				
Age	≥ 75 years	29/37	2.3 (1.4–3.6)	0.001	2.1 (1.3–3.4)	0.002	38/47	2.3 (1.5–3.5)	<0.001	2.0 (1.3–3.1)	0.002
	<75 years	46/88					63/121				
Operation	Hartmann	7/7	2.8 (1.3–6.3)	0.007	–	–	13/13	3.5 (2.0–6.4)	<0.001	2.4 (1.3–4.5)	0.006
	AR or APE	68/118					88/155				
Tumour size	< 5 cm	43/71	1.1 (0.7–1.8)	0.589	–	–	59/98	1.0 (0.7–1.5)	0.986	–	–
	≥ 5 cm	32/54					42/70				
Pathological type	Mucinous or signet ring	4/6	0.9 (0.3–2.4)	0.771	–	–	3/5	0.8 (0.3–2.6)	0.744	–	–
	Adenocarcinoma	71/119					98/163				
Grade	High	7/13	1.0 (0.5–2.2)	0.978	–	–	16/24	1.5 (0.9–2.5)	0.152	–	–
	Low/average	68/112					85/144				
Venous invasion	Present	11/18	1.4 (0.7–2.7)	0.299	–	–	19/33	1.0 (0.6–1.7)	0.909	–	–
	Absent	64/107					82/135				
Free serosal surface involved	Yes	2/4	0.8 (0.2–3.4)	0.806	–	–	3/5	1.2 (0.4–3.9)	0.717	–	–
	No	73/121					98/163				
Adjacent organ infiltrated	Yes	75/125	–	–	–	–	2/2	1.8 (0.4–7.4)	0.393	–	–
	No	0/0					99/166				
uPAR in tumour epithelial cells	Weak, moderate & Strong (i.e., Positive)	51/73	2.0 (1.2–3.3)	0.006	1.9 (1.1–3.1)	0.014	–	–	–	–	–
	vs.										
	Negative	24/52									
	Strong	–	–	–	–	–	43/63	1.6 (1.1–2.4)	0.017	1.6 (1.1–2.3)	0.031
	vs.										
	Negative, weak & moderate						58/105				

BHR: Bivariate Hazard Ratio, MHR: Multivariable Hazard Ratio, AR: Anterior resection, APE: Abdominoperineal excision

**Invasive front**. For stages B and C combined, there was no significant association between uPAR^E^ and overall survival (p = 0.179). Patients with stage C tumours had no association (p = 0.848) but the *p*-value approached significance in stage B (p = 0.087). In case the latter result was a function of small sample numbers in some categories, the data were re-examined by combining negative, weak and moderate staining, as their association with overall survival did not differ significantly (p = 0.294). Kaplan Meier analysis of uPAR^E^ expression in the combined group showed patients with strong expression of uPAR^E^ had a significantly poorer survival (p = 0.017) (Fig. 3b). This persisted after multivariable analysis adjusting for other prognostic variables (HR 1.5 [95% CI 1.1–2.3] Wald-p 0.031) (Table 2).

### uPAR^S^ at the invasive front and survival in stage C tumours

Evaluation of uPAR expression in RC stroma-associated cells employed two different approaches. Firstly, overall stromal staining intensity was scored as 0, 1, 2 and 3 (Fig. 2b), in the same way as uPAR^E^. Secondarily, the presence of peritumoural accentuation (uPAR^PT^; uPAR expression in stroma but concentrated around the tumour cells) was compared with its absence (Fig. 2c).

**Central region**. Overall survival was not significantly related to uPAR^S^ either for stages B and C combined (p = 0.492), stage B alone (p = 0.071) or stage C alone (p = 0.436). The presence or absence of uPAR^PT^ in the central tumour showed no significant association with patient survival.

**Invasive front**. uPAR^S^ was not significantly associated with overall survival for combined stages B and C (p = 0.226) or stage B alone (p = 0.641). However, within stage C, there was an overall tendency towards increasing survival as uPAR^S^ expression progressed from negative to strong (p = 0.015). There was no survival significance between negative and moderate, whereas there was a significant difference between moderate and strong (p = 0.031). Therefore negative to moderate staining were combined into a single category and compared to strong expression. Strong uPAR^S^ expression was significantly associated with longer overall survival (Fig. 3c; p = 0.009). After adjustment for other prognostic variables this difference persisted (HR 0.6 [95% CI 0.4–0.9] Wald-p = 0.007) (Table 3). Furthermore the stage C patient group with uPAR^PT^ present in the invasive front of tumour tissues also showed a longer survival time compared with patients without uPAR^PT^ (Fig. 3d; p = 0.017) on multivariable analyses (HR 0.7 [95% CI 0.5–0.9] Wald-p 0.016) (Table 3).

**Table 3:** Association between stage C patient overall survival and expression of uPAR in stroma-associated cells (detected by R4 MAb) in invasive front of tumour tissues with adjustment for other potentially prognostic variables.

Variable	Category	Deaths/Total	BHR (95% CI)	Wald p	MHR (95% CI)	Wald p
Sex	Male	87/113	1.2 (0.9–1.8)	0.245	–	–
	Female	46/66				
Age	≥ 75 years	41/47	1.6 (1.1–2.4)	0.009	1.7 (1.2–2.5)	0.007
	<75 years	92/132				
Operation	Hartmann	8/9	3.1 (1.5–6.4)	0.001	4.5 (2.1–9.5)	<0.001
	AR or APE	125/170				
Tumour size	< 5 cm	55/77	1.0 (0.7–1.4)	0.928	–	–
	≥ 5 cm	78/102				
Pathological type	Mucinous or signet ring	12/15	1.1 (0.6–2.0)	0.724	–	–
	Adenocarcinoma	121/164				
Spread beyond muscularis propria	Yes	111/150	1.3 (0.8–2.1)	0.284	–	–
	No	21/29				
Apical node involved	Yes	8/9	2.8 (1.4–5.8)	0.004	–	–
	No	125/170				
≥ 4 nodes involved	Yes	50/63	1.4 (1.1–2.1)	0.039	–	–
	No	83/116				
Node ratio	≥ 40%	32/38	1.8 (1.2–2.7)	0.003	1.8 (1.2–2.8)	0.005
	<40%	101/141				
Grade	High	49/67	1.3 (0.9–1.9)	0.128	–	–
	Low/average	84/112				
Venous invasion	Present	53/63	1.8 (1.2–2.5)	0.001	1.5 (1.1–2.2)	0.019
	Absent	80/116				
Free serosal surface	Yes	10/15	0.9 (0.5–1.8)	0.819	–	–
involved	No	123/164				
Adjacent organ infiltrated	Yes	6/6	3.5 (1.5–8.1)	0.002	3.0 (1.3–6.9)	0.012
	No	127/173				
uPAR in stroma-associated cells	Strong	30/47	0.6 (0.4–0.9)	0.009	0.6 (0.4–0.9)	0.007
	vs.					
	Negative, Weak & Moderate	103/132				
uPAR accentuated in	Positive	55/85	0.7 (0.5–0.9)	0.017	0.7 (0.5–0.9)	0.016
peritumour	Negative	78/94				

BHR: Bivariate Hazard Ratio, MM HR: Multivariable Hazard Ratio, AR: Anterior resection, APE: Abdominoperineal excision.

### uPAR at the adjacent non-neoplastic mucosal tissues

For epithelia uPAR expression at the adjacent non-neoplastic mucosal tissues (n = 312), 36 cases demonstrated strong uPAR expression (19 in stage B and 17 in stage C), 58 cases had moderate expression (28 in stage B and 30 in stage C), 81 cases had weak expression (41 in stage B and 40 in stage C) and 137 cases were negative (68 in stage B and 69 in stage C). In stage B, there was no association between staining intensity and survival (p = 0.700) while in stage C there was no difference in survival between negative, weak and intermediate. The only significant association was longer survival for strong than for negative (p = 0.018) but, as this was based on only 17 cases with strong staining, it appeared anomalous and may be a Type 1 error arising from this small number. uPAR expression in stroma was almost absent, with negative expression in 320 cases (156 in stage B and 164 in stage C) and only moderate expression in 9 cases (6 in stage B and 3 in stage C), insufficient uPAR expression specimens were available for a comprehensive statistical analysis.

## Discussion

Although the prognostic relevance of uPAR in cancer has been extensively studied, significant discrepancies have rendered much of the work inconclusive. Two major issues remain unresolved: firstly, the discrepancy regarding the cell types where uPAR is overexpressed (i.e., uPAR^E^ or uPAR^S^), and secondly, the prognostic relevance of uPAR in different cell types and different stages of tumour progression. In this study we have addressed the first paradox by demonstrating that uPAR expression in different cell types can be detected using two epitope-specific anti-human uPAR MAbs #3937 and R4. These antibodies delineated between uPAR^E^ and uPAR^S^ expression in RC tissues, showing antigen expression could be differentially detected in different cell types and tumour locations in the same RC tissues. Upon examination of uPAR^E^ and uPAR^S^ from 349 stage B or C RC tissues, we were able to decipher the second controversy, revealing that elevated uPAR^E^ in both the central region and invasive tumour front adversely correlated with stage B overall survival, whereas elevated uPAR^S^ at the invasive front favourably correlated with stage C overall survival.

The recognition of different uPAR epitopes by different antibodies is an important factor to be considered, not only for detection in different cell types but also for determination of the potential clinical prognostic relevance of uPAR. There are multiple anti-uPAR polyclonal antibodies (PAbs) and MAbs which have been developed and studied extensively in clinical applications [6]. Of these, MAbs #3937 (like #3936) and R4 (like R2) are most frequently used for uPAR^E^ and uPAR^S^ detection respectively and stand at the centre of disparate results obtained by different laboratories. Several factors may explain the specificity of these different antibodies, the primary one being binding to different “available” epitopes reflecting potentially diverse roles of uPAR in each cell type. As uPAR has 42 known interacting partners [5], it is also possible that the antibody epitopes may be masked by other uPAR interacting partners in different cell types. The multifunctional nature of uPAR is a function of its interactome [3,5], and therefore uPAR detected by different epitope-specific MAbs may have different interacting partners, which may reflect divergent functions in discrete cell types. This concept is supported by the fact that the population of soluble uPAR (suPAR) in specific cell types has shown diametrically altered staining patterns reflecting functional differences as a result of structural variations [25]. suPAR has been found in three different forms in both tissues and body fluids [26] depending upon the number of domain(s) present (e.g. D1D2D3, D2D3 or D1) [25]. suPAR_D1D2D3_ has been proposed as a uPA-scavenger, and although it has the ability to bind uPA, the protease does not autocleave the linker region between D1 and D2 [25,27]. Therefore, increased suPAR_D1D2D3_ may reduce uPA-dependant proteolysis leading to inhibition of cancer metastasis by reducing the ability of cells to leave the ECM or drive alternative uPAR-dependant biologies [25]. In contrast, suPAR_D2D3_ acts primarily as a chemotactic agent promoting an immune response via the SRSRY sequence in the D1 & D2 linker-region [25]. Furthermore, other studies have demonstrated a high concentration of suPARs (i.e., suPAR_D1_ and suPAR_D1D2D3+D2D3_) in CRC patient sera associated with significantly reduced overall survival [28]. Therefore, based on our evidence, uPAR^E^ and uPAR^S^ may reflect functional differences in the biology of uPAR expressed in cell types that differently influences tumour biology, cancer metastasis and therefore association with patient survival. To address these possibilities, survival significances for both uPAR^E^ and uPAR^S^ at both the central region and the invasive tumour front between stages B and C were analysed (i.e., detected by epitope-specific MAbs in different cell types, in different locations of tumour progression, and at different cancer stages).

Not only do our results confirm previous studies demonstrating that both uPAR^E^ and uPAR^S^ concentrate at the invasive tumour margin in CRC, they also demonstrate that uPAR^E^ adversely correlates with patient survival in RC. Thus, collectively it appears that uPAR^E^ may be an independent negative prognostic indicator of survival in many types of human cancers, including CRC [6]. This was especially apparent in a recent large CRC study (n = 811) which demonstrated that uPAR^E^ was significantly associated with poor survival across all CRC stages (i.e., stages A-D) [15]. We were particularly interested in stages B and C RC patients in our study, because, although these patients are deemed to have had a curative operation, there remains a pronounced stage difference in their survival [2]. Interestingly, for both central and frontal tumours, survival significance of uPAR^E^ appeared only in RC stage B tissues. The results indicate that uPAR^E^ may be a prognostic survival indicator for pre-lymph node metastatic tumours, demonstrating an independent significance in multivariable analyses. Additionally, uPAR^E^ intensity in central and invasive tumour fronts was differentially associated with survival. In the central region, positive uPAR^E^ (strong, moderate & weak) had significantly shorter survival compared to negative, whereas in the invasive front, strong uPAR^E^ had shorter survival compared to a combined group (moderate, weak and negative). We propose this observation could be due to expression of uPAR^E^ in the frontal tumour region, facilitating metastasis to neighbouring tissues or lymph nodes. Indeed, our results, and those from others, demonstrate that uPAR^E^ was concentrated in the invasive front [6,7,9,10,29]. Furthermore, uPAR has been shown to play a crucial role in cancer cell invasion and metastasis involving many biological processes including epithelial-mesenchymal transition (EMT), ECM degradation, cell migration and adhesion, and activation of MAP kinase and Ras pathways [3], all supporting the presence of uPAR in the invasive tumour front. Overall, our results provide an understanding of uPAR^E^ distribution in the tumour microenvironment and show that uPAR^E^ is a survival indicator of non-metastatic RC tumours (i.e. stage B) and its expression should be considered in both the central region and invasive front of tumours in any future diagnostic, prognostic and/or therapeutic studies.

Unlike uPAR^E^, uPAR^S^ remains controversial in terms of survival significance. The two most recent studies demonstrated that uPAR^S^ was negatively associated with CRC patient survival. Boonstra *et al*., [17] demonstrated that uPAR^S^ was adversely associated with overall survival as well as DFS across all stages from A to D (n = 262). Illemann *et al*. [30] also demonstrate uPAR positive macrophages in tumour cores (stroma-associated cells in the central tumour region) were negatively associated with overall survival in all stages (n = 244), but the significance did not appear in the tumour invasive front. Conversely, our study shows that uPAR^S^ was positively associated with overall survival specifically in stage C at the invasive front only, supported by independent significance in multivariable analysis. In terms of MAb utilisation, Boonstra *et al*., used ATN615 [17] whilst Illemann *et al*., used rabbit PAb and R2 MAb (with identical staining patterns observed for PAb & R2 MAb) [30], whereas our study utilised R4 MAb. The characteristics of ATN615 (together with ATN658) have been extensively studied recently [17,31–34]. Specifically, uPAR epitope-binding sites for these MAbs were identified from the crystal structure [31,32]. For ATN615, the P189 and R192 of human uPAR D3 region were identified as critical to formation of the epitope [32]. The epitope sites for R2 and R4 (D275 and L276 (for MAb R2) and R192, D214, G217 and S269 (for MAb R4) in the D3 region of uPAR) have also been reported by other studies using surface plasmon resonance and/or Western blotting [35,36], although no crystal structure studies are currently available. It is likely that these MAbs are targeting the D3 region of uPAR but through different epitopes located in this domain. Thus the different staining patterns of these MAbs may represent different roles of uPAR or different uPAR-interactomes, linked to differential survival significance results. The crystal structures of both the uPAR-R4 and uPAR-R2 complexes may be an important direction to pursue in the future.

Importantly, Illemann *et al*., [30] showed that the association of uPAR^E^ and uPAR^S^ with survival significance was independent of CRC stage, which was further confirmed in our study. These data suggest that the plasminogen activation proteolytic cascade is not only implicated in tumour cell invasion/metastasis but is also related to patient survival rates at different CRC stages. However, the difference between Illemann’s study and ours is the opposite prognostic relevance of uPAR^S^ at the different location during tumour progression (i.e., negative prognostic indicator in central region *vs*. positive prognostic indicator in invasive front, respectively). In CRC tumour-associated stroma, expression of uPAR has been observed in monocytes/macrophages, fibroblasts, neutrophils, myofibroblasts and endothelial cells [9,14,17,29]. Of these, macrophages (also known as tumour-associated macrophages (TAMs)) are a major source of uPAR expression [29] and are the most abundant immune cells in the tumour microenvironment [37]. During tumour progression, circulating monocytes in blood vessels are recruited to the tumour site and differentiated into mature macrophages such as M1- and M2-polarised macrophages [38]. M1-macrophages are known to mediate tumour elimination, whilst M2-macrophages have a rather contradictory role as acting in either a pro- or anti-tumour fashion [37]. In the tumour microenvironment, TAMs resemble M2-macrophages and induce the production of a large range of growth factors and proteolytic enzymes such as EGF, TGFβ1, VEGE and MMPs to stimulate ECM degradation, thus promoting tumour metastasis, resulting in poor cancer prognosis [37]. However other studies have demonstrated that TAM accumulation at the invasive tumour front can also be associated positively with CRC patient survival [39–41]. Furthermore, supporting data has demonstrated that TAM concentrated around tumour cells are able to induce apoptosis in a Fas ligand-dependent manner, and the degree of apoptotic cancer cells is inversely correlated with haematogenous metastasis, emphasising the protective role of TAMs [42]. These results suggest that the location of TAMs in CRCs (i.e., at invasive front or peritumourally) appear to be an important factor in antitumour activation. This concept is further supported by other reports demonstrating that peritumoral macrophages are likely to have less contact with tumour-derived cytokines, and are positioned in less hypoxic areas, indicating that they may display a tumouricidal rather than tumour promoting activity [37]. This model very closely aligns with our data that demonstrates uPAR^S^ is a positive prognostic indicator of survival in the invasive front of stage C RCs. These data are further supported by the presence of uPAR^PT^ at the same location and stage, which was associated with longer survival than when uPAR^PT^ was absent. Collectively, it is possible that the population of M1 macrophages and/or newly recruited monocytes (i.e., before polarisation) may represent a higher proportion of macrophages at the invasive front and in peritumoural regions of CRCs, and that uPAR^S^ detected by R4 might be expressed by those stroma-associated cells. In fact, a recent study demonstrated that in an experimental model of colitis, uPAR controls the function of intestinal macrophages by reducing inﬂammatory cytokines and controlling M1 and M2 polarisation [43]. For future studies, simultaneous IHC of monocytes, M1- and M2-polarised macrophages, and R4 (or other MAbs such as ATN615 or R2) on serial CRC TMA sections may further clarify our understanding of the role/s of uPAR in stroma-associated cancer biology.

Although uPAR^E^ was expressed in a significant number of adjacent non-neoplastic mucosal tissues, we have not considered to use this as an internal standard because it may not be as representative as healthy mucosa, since the histologically normal tissues used in this study were taken from 1–2cm from the tumour margin. We have recently demonstrated that integrin ανβ6 (a potential prognostic indicator of colorectal cancer and recognised to be absence or low expression in normal tissues) was expressed in almost all histological normal mucosa (from same TMA used in this study) [44]. As the integrin ανβ6 is one of key regulators of EMT along with TGF beta1 [45,46], it indicates that EMT-associated changes are occurring in that tissue. This observation also supported by other types of cancer demonstrated that the expression of EMT markers (e.g., α-smooth muscle actin & SNAIL) occur in “apparently” histologically normal breast tissue that is located 1cm away from breast cancer tissue margins [47].

In conclusion, we have found that uPAR^E^ is associated with poorer RC survival in stage B (in the central and the invasive front regions) whilst uPAR^S^ is correlated with longer survival in stage C (in the invasive tumour front). This indicates that uPAR has an opposite role in different cell types at different tumour locations across RC stages B and C. We have proposed that these functional differences may potentially be related to differences in the uPAR-interactomes present in distinct cell types. In this regard, we have already unequivocally shown uPAR interacts with αvβ6 which is an epithelially-restricted integrin (i.e., one that would never occur in stromal cells) [48]. Therefore, a comprehensive study of the uPAR interactome in different cell types and consequent reactivity of uPAR with various anti-uPAR MAbs is a necessary step towards an understanding of its roles in CRC. Indeed, MAb inhibition of the uPAR-integrin interactome has been recently proposed as a new anti-cancer therapeutic approach and a basis to develop tumour imaging methodologies [31,34,49,50]. Overall, accurate prediction of patient survival based on uPAR expression coupled with a better understanding and targeting of specific uPAR interactomes (using human or humanised MAbs against interaction surfaces, inhibitory peptides or small molecule antagonists of interactomes) may lead to the development of novel, personalised companion immunopathology prognostics and anti-metastasis therapeutics.
